# Genetic contributions to attentional response time slopes across repeated trials

**DOI:** 10.1186/s12868-015-0201-3

**Published:** 2015-10-15

**Authors:** Rebecca A. Lundwall, James L. Dannemiller

**Affiliations:** Brigham Young University, Provo, UT USA; Department of Psychology, MS-25, Rice University, P. O. Box 1892, Houston, TX 77251-1892 USA

**Keywords:** Exogenous orienting, Reflexive attention, Candidate gene, RT slope, Linear trend

## Abstract

**Background:**

Attention provides vital contribution to everyday functioning, and deficits in attention feature in many psychological disorders. Improved understanding of attention may eventually be critical to early identification and treatment of attentional deficits. One step in that direction is to acquire a better understanding of genetic associations with performance on a task measuring reflexive (exogenous) visual attention. Reflexive attention is an important component of overall attention because (along with voluntary selective attention) it participates in determining where attention is allocated and how susceptible to distractors the subject might be. The task that we used involves the presentation of a target that is preceded by one of several different types of cues (none, double, or single, either ipsilateral or contralateral to where the target subsequently appears). We used several different outcome measures depending on the cue presented. We have previously studied the relationship between selected genes and mean response time (RT). Here we report on the contributions of genetic markers to RT increases or decreases over the course of the task (linear trend in RT slope).

**Results:**

Specifically, we find that RT slope for a variety of reflexive attention outcome measures is dependent on *DAT1* genotype. *DRD4* was near significant for one outcome measure in the final (best) model. *APOE*, *COMT*, and *DBH* were not significant in any models.

**Conclusions:**

It is especially interesting that genotype predicts linear changes in RT across trials (and not just mean differences or moment-to-moment variability). *DAT1* is a gene that produces a protein involved in the transport of dopamine from the synapse. To our knowledge, this is the first study that has associated neurotransmitter genotypes with RT slope on a reflexive attention experiment. The direction of these effects is consistent with genetic risk for attention deficit hyperactivity disorder (ADHD). That is, those with two risk alleles for ADHD (6R/6R on the *DAT1* intron 8 VNTR) either got slower as the task progressed or had the least improvement. Those with no risk alleles (5R/5R) had the most improvement in RT as the task progressed.

## Background

**Fig. 1:**
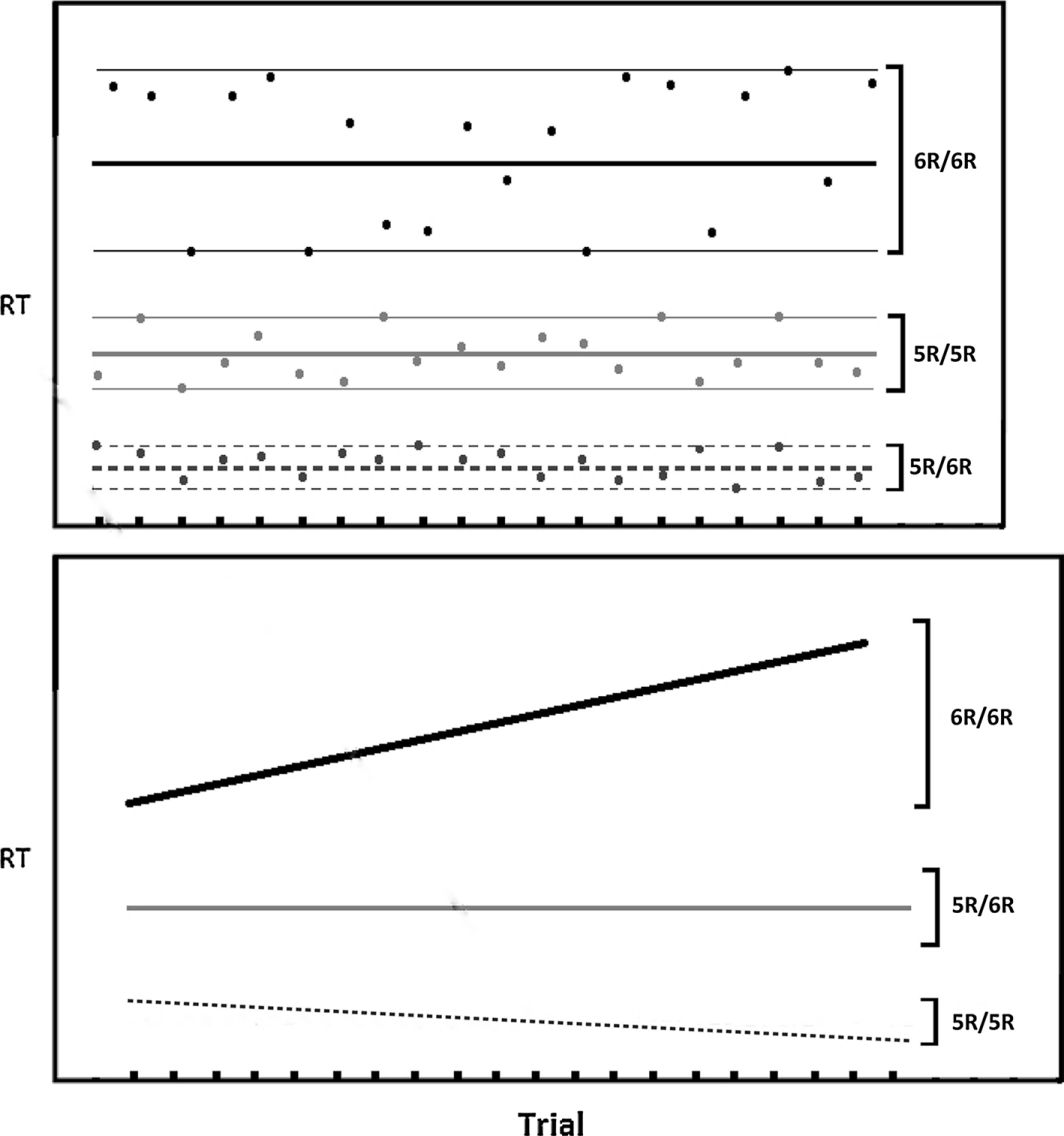
Hypothetical illustration of two types of variability. The top portion shows three individuals who differ in the spread (moment-to-moment variability) around a regression line after slope is covaried out. The bottom portion represents variability of RT slope between individuals. Some individuals become faster (or slower) over the 20 presentations of a given trial type. We are primarily interested in RT slope variability in this paper

Attention is a broad concept that has often been divided in the literature along various dimensions. One such division involves reflexive (exogenous) versus sustained attention. Reflexive attention refers to a stimulus-driven reorienting of the brain’s resources, often to an external object that newly appears, has a relatively salient color, or involves motion. The cued-orienting task that we used in our first study [1] is an example of a reflexive task and is similar to that used by Posner et al. [2]. Stimuli flash briefly on the computer display and subjects automatically move their attention. It is generally assumed that attention has been captured reflexively if subjects were faster at responding to a target that was preceded by the presentation of a brief pre-cue at that same location. Attention can be captured in this way even though the stimulus presentation is too brief to involve eye movements. On the other hand, sustained attention is measured with an effortful task designed to require vigilance over time. For example, the Continuous Performance Test (CPT) [3] and the Sustained Attention to Response Task (SART) [4] involve the presentation of a stream of stimuli, some of which require a response while others require that a response be withheld. Examining slope in response time (RT) over trials (see Fig. 1) on a reflexive attention task involves some elements of reflexive attention and some elements of sustaining attention to the task over time (~20 min). Contributions of reflexive attention to attentional deficits are often overlooked [5–8] but could be useful for a more complete understanding of disorders that have an attentional component, such as ADHD, autism, anxiety, and depression by answering questions about biological contributions to attention using exogenous versus endogenous cues [9, 10]. For example, one question might be whether it is the nature of the task itself (i.e., designed to induced effortful attention) that induces declining performance over time or if non-effortful (reflexive) attention tasks can induce the same decline in performance in some individuals. There are several studies that discuss genetic associations with RT changes across the course of a sustained attention task [11–14]; however, the literature contains almost no genetic-association studies on how RT changes across trials during the course of a reflexive task. Such studies might be helpful in determining if declining performance over time shares similar or has distinct genetic influences in reflexive attention and sustained attention tasks. The genes we selected are related to the availability of neurotransmitters, such as acetylcholine and dopamine (see Table 1). We have previously found some of these genes to be associated with mean RT difference scores [1]. In this paper, we extend those findings with a novel look at the influence of these genes on the slope of RT over the course of the 20-min reflexive attention task. Looking at RT slope is unlike looking at moment-to-moment RT variability because the former is probably dependent on alertness or learning [15, 16] while the latter is probably related to vigilance and the ability to detect a signal beyond neural noise [17, 18].

**Table 1:** Rationale for genetic markers selected

Genetic marker	Risk allele	Biological effect	Cognitive associations
*COMT* rs4680	G	G at rs4680 produces valine making the gen product more active in catabolizing dopamine [59]	Reduced cognitive function generally [59] and ADHD [60]
*DAT1* intron 8 VNTR	6R	6R leads to more dopamine transporter, less dopamine in the synapse [61] and thus terminates the signal [37]	Increased RT costs for targets in the left hemifield [62] and ADHD [63]
*DRD4* rs747302	C	C leads to fewer dopamine receptors via reduced transcription [64]	Association with ADHD [64]
*APOE* rs429358 + rs7412	ε4	ε4 reduces acetylcholine receptors [65] and may diminish synthesis of acetylcholine [31]	Reduced spatial cueing in visual tasks for middle aged (non-demented) carriers of the ε4 allele [34, 66, 67]
*DBH* rs1108580	A	DβH converts dopamine to norepinephrine, therefore, the A allele is associated with lower norepinephrine to dopamine ratios [68]	Association with ADHD [69, 70]

Bellgrove et al. [62] refer to 3R but according to NN Rommelse, ME Altink, A Arias-Vasquez, CJ Buschgens, E Fliers, SV Faraone, JK Buitelaar, JA Sergeant, B Franke and J Oosterlaan [64] 3R is now called 6R

Candidate gene studies that show associations between specific genes and various attentional measures provide evidence of genetic influences on attention. Such an association might occur because a given gene influences the availability of a neurotransmitter. That is, the biological pathway from gene to behavior could include neurotransmitters whose availability impacts those behaviors. For example, attention [19, 20] and memory [21] are known to be influenced by the availability of the neurotransmitters. The varying availability of these neurotransmitters due to genetic differences could make behaviors like attending more or less efficient in some individuals, and this, in turn, could be captured by various measures of attention.

One component of ADHD is difficulty in maintaining attentional arousal. Individuals with ADHD are frequently identified as having this difficulty [11, 12, 22–29]. Usually the connection between maintaining arousal and dopamine is made for moment-to-moment variability. For example, Johnson et al. [30] make the argument that dopamine must be involved in moment-to-moment RT variability because methylphenidate (which increases dopamine availability) reduces RT variability. Exaggerated RT variability could arise because of difficulty in sustaining attention such that RTs rise and fall over the course of a task independently of the task requirements. RT variability may increase with less dopamine availability because it becomes harder to distinguish signal from noise in the nervous system. That is, reduced availability of dopamine could lead to weaker neural signals giving random neural noise a larger impact on target detection and response [17, 18].

Another form of variability over trials would be monotonic increases or decreases in RT. That is, dopamine availability could also be related to RT slope over the course of a task. For example, Bioulac et al. [23] found that children with ADHD decline in performance over time on a Continuous Performance Test. This finding could apply to slight attentional deficits found in nonclinical populations as well. In such populations, there may be variations in neurotransmitters that have been associated with attention generally and these variations could contribute to an increase in RT over the course of a task.

This is interesting because our previous analysis of the data in the current study showed that individuals from the general population differed on their mean RTs depending on their neurotransmitter-related genotypes [1]. Risk alleles for each genetic marker were identified in previous studies as associated with ADHD, greater RT costs, and/or reduced cognitive functioning generally. Each genetic marker was coded such that zero indicates neither parent contributed a risk allele for these cognitive outcomes, one indicates one parent contributed a risk allele, and two indicates that each parent contributed a risk allele. Those findings that used an RT attention task generally associated genetic markers with mean RTs or RT difference scores. As mentioned previously, we could find no studies in the literature on genetic associations with RT slope in reflexive attention tasks.

In the current study we asked a simple question: Are RT slopes on a reflexive attention task associated with specific genes? We have genotyping information for subjects on the dopamine-related genes *COMT*, *DAT1*, *DBH* and *DRD4*. We also have information on *APOE*, which carries risk for Alzheimer’s disease, is related to acetylcholine and has been related to cued orienting, even in young adults and adolescents [31–33] and with attention generally [34, 35]. It is useful to examine genes that do not fit with the dopamine hypothesis of attentional deficits and yet have a logical and historical association with attention. Using these data previously on some of these genes [1], we found significant mean RT differences between individuals with different genotypes. Our goal was to determine if there are also genetic associations with RT slopes across trials in an reflexive task. Thus, we are looking for gene-by-trial interactions by modeling logarithm transformed RT as the dependent variable (the outcome in the multilevel model). Such results would be the first to our knowledge to demonstrate such associations. Our interest in RT slope is to give a more complete picture of what is happening in the minds of those individuals who struggle with attentional deficits, which could potentially lead to better environmental supports and treatments.

## Results

All markers were in Hardy–Weinberg equilibrium (*P*s > 0.90). In the following models, we were specifically interested in gene-by-trial interactions because they signal differences in the response time trends across trials depending on genotype (see Table 2 for correlations between covariates, genetic markers, and the dependent variable.).

**Table 2:** Correlations between the dependent variable, genetic markers, and covariates

	1	2	3	4	5	6	7	8	9	10	11	12	13	14
1. Log RT		0.36**	−0.17	0.11	−0.05	0.01	−0.08	−0.20*	0.07	0.09	0.15	0.10	0.13	0.07
2. SD of Log RT			−0.02	0.18*	−0.01	0.05	−0.11	−0.23**	0.06	0.10	0.20*	0.07	−0.29**	0.38**
3. APOE				−0.10	−0.15	0.00	−0.09	0.00	−0.03	0.11	−0.03	0.04	−0.05	0.02
4. COMT					−0.08	−0.03	0.09	−0.25**	0.16	0.15	0.08	0.13	−0.06	0.14
5. DAT1						−0.06	−0.14	0.06	0.08	−0.24**	−0.02	0.19*	0.05	−0.03
6. DBH							−0.02	−0.19*	0.22**	−0.07	0.08	0.05	−0.19*	−0.03
7. DRD4								0.21*	−0.11	−0.07	−0.13	−0.02	0.13	−0.04
8. Caucasian									−0.63**	−0.32**	−0.53**	−0.02	0.29**	−0.08
9. Asian										−0.10	−0.17*	−0.06	−0.21*	0.02
10. Black											−0.08	0.01	−0.08	0.07
11. Hispanic												0.10	−0.12	0.05
12. Sleepiness													0.00	0.02
13. Age														−0.11
14. Error rate														

Note that the highest correlation with log transformed RT is the standard deviation of log transformed RT. This is likely due to the mathematical relationship between SD of log RT and log RT

** Significant at *p* < 0.01

* Significant at *p* < 0.05

Multiple models were tested for each outcome measure (cue-target condition). We began with a full model (including all predictors and covariates) and compared subsequent models for improved model fit (as determined by a reduction in Bayesian Information Criteria; BIC). Full models used logarithm transformed RT as the dependent variable and included predictors of interest (*APOE*, *COMT*, *DAT1*, *DBH*, *DRD4*, and trial number), covariates (the standard deviation of logarithm transformed RT, error rate, dummy coded ethnicity, side of target, sleepiness score, and age), and the interactions of interest (the slopes, which are the five gene-by-trial interactions). The final (reduced model) eliminated all covariates except ethnicity. We also tested models removing non-significant interactions, but these did not improve model fit for any cue-target condition. However, BIC did improve and the same gene-by-trial interactions (the slopes) were significant or nearly significant when using a simpler model without including covariates (the standard deviation of the log transformed RT, target side, error rate, sleepiness, or age). For parsimony, we settled on the simpler models which showed substantially the same results and had significantly smaller BIC values (as determined by χ^2^ deviance statistics). For every cue-target condition, these were the best models and the improvements over models with all predictors (the full models) were significant at *p* < 0.001. Reporting Akaike’s Information Criterion (AIC) instead of BIC does not change the model building results in any instance.

Estimates from tested models are presented in Tables 3, 4, 5, 6, 7, 8, 9, 10 and 11 (one table per cue-target condition).^1^ Note that statistical significance was determined using log_10_(RT) as the dependent variable, while the parameter estimates in Tables 3, 4, 5, 6, 7, 8, 9, 10 and 11 have been retransformed to the original RT scale to aid in conceptual clarity.^2^ Additionally, Table 12 shows slopes for the different genotypes from a simple regression of untransformed RT on trial for each genotype to aid in conceptual clarity. The net change in RT across 200 trials can be derived from the slopes in Table 12 by multiplying the slope by 200 trials. For example, for the DAT1 5R/5R genotype for the *Single Dim Valid* condition: mean(RT change) = 200 trials × (−0.56) ms/trial = −112 ms across 200 trials (subjects with this genotype responded more than 100 ms faster on trials at the end of the experiment than at the beginning). Our primary interest is in whether different genotypes showed non-zero slopes (RT across trial). While an additional slope was significant with the full model (*DAT1* on *Single Bright Valid*), the reduced models had a lower BIC, and so they are described here.

**Table 3:** Estimates from models of the predictors of log-RT for the dual by bright condition

Parameter	Empty model		Full model		Non-significant interaction removed		Covariates removed	
	Mean	SE	Mean	SE	Mean	SE	Mean	SE
Fixed effects								
Intercept	466.98**	1.58	273.63**	17.67	269.35**	14.91	433.08**	13.48
Level 1 (trial-specific)								
Trial			−0.04	0.11	−0.01	0.05	−0.04	0.05
Side			−1.98**	1.59	−1.99**	1.60		
Level 2 (individual)								
Age			0.95**	0.19	0.95**	0.19		
Asian			15.14**	4.90	15.16**	4.90	11.99**	2.22
Black			22.05**	9.68	22.08**	9.69	24.30**	4.49
Hispanic			4.94*	5.37	4.94	5.37	9.94**	2.39
White			0^a^	0^a^	0^a^	0^a^	0^a^	0^a^
Error rate			−299.52**	155.79	−299.90**	155.87		
Sleepiness (centered)			0.29	0.50	0.29	0.50		
Std. Dev. of Log RT			1124.44**	1548.55	1123.96**	170.42		
APOE (0 risk)			5.63	12.82	7.18**	6.42	10.95	5.94
(1 risk)			8.05*	8.11	9.85**	4.21	5.57	3.76
(2 risk)			0^a^	0^a^	0^a^	0^a^	0^a^	0^a^
COMT (0 risk)			−11.05**	10.99	−3.16	5.67	−13.25*	5.06
(1 risk)			4.12	8.08	8.78**	4.23	1.51	3.70
(2 risk)			0^a^	0^a^	0^a^	0^a^	0^a^	0^a^
DAT1 (0 risk)			30.71**	17.51	22.85**	8.95	17.87*	8.04
(1 risk)			3.00	6.94	−1.62	3.53	4.57	3.20
(2 risk)			0^a^	0^a^	0^a^	0^a^	0^a^	0^a^
DBH (0 risk)			3.06	9.78	3.20	5.05	4.72	4.51
(1 risk)			−7.27*	7.80	−5.83**	3.98	−4.17	3.59
(2 risk)			0^a^	0^a^	0^a^	0^a^	0^a^	0^a^
DRD4 (0 risk)			11.49**	9.26	10.91**	8.95	11.34**	4.29
(1 risk)			14.03**	8.03	13.43**	7.77	15.94**	3.72
(2 risk)			0^a^	0^a^	0^a^	0^a^	0^a^	0^a^
APOE (0 risk)*Trial			NS	NS			NS	NS
COMT (0 risk)*Trial			NS	NS			NS	NS
DAT1 (0 risk)*Trial			NS	NS			NS	NS
DBH (0 risk)*Trial			NS	NS			NS	NS
DRD4 (0 risk)*Trial			−0.09*	0.08	−0.08*	0.08	−0.09*	0.04
(1 risk)			−0.04	0.07	−0.03	0.07	−0.04	0.03
(2 risk)			0^a^	0^a^	0^a^	0^a^	0^a^	0^a^
Covariance parameters								
Repeated measures			5519.27**	0.16	5525.59**	0.16	6344.33**	0.18
Number of parameters	2		31		23		26	
Schwarz’s Bayesian criterion (BIC)	−6453.45		−5096.70		−5223.97		−4837.26	

Significant values for ethnicities indicate that the ethnic group is different from Caucasians on RT values. Other significant values indicate that the estimate is different from zero. We report in the “Results” section tests of fixed effects for gene by trial interactions to determine if slopes differ by genotype

Note that this table shows model parameter estimates when analyzing log transformed RTs. The values have been retransformed. However, to get a rough idea of slope differences by genotype group, please see Table 12 for raw RTs (ms)

*NS* the slopes were not significantly different from zero and so the row has been collapsed

* Significant at *p* < 0.05

** Significant at *p* < 0.01

^a^This parameter has been set to zero

**Table 4:** Estimates from models of the predictors of log-RT for the dual by dim condition

Parameter	Empty model		Full model		Non-significant interaction removed		Covariates removed	
	Mean	SE	Mean	SE	Mean	SE	Mean	SE
Fixed effects								
Intercept	491.83**	1.70	267.25**	18.52	271.83**	15.10	454.62**	14.37
Level 1 (trial-specific)								
Trial			−0.03	0.11	−0.06**	0.03	−0.03	0.05
Side			−1.98**	1.67	−1.98**	1.68		
Level 2 (individual)								
Age			1.24**	0.20	1.24**	0.20		
Asian			9.05**	5.16	9.06**	5.16	5.43*	2.39
Black			24.40**	10.05	24.37**	10.06	27.33**	4.76
Hispanic			2.94	5.58	2.95	5.59	8.24**	2.53
White			0^a^	0^a^	0^a^	0^a^	0^a^	0^a^
Error rate			−306.56**	164.62	−304.52**	164.75		
Sleepiness (centered)			0.21	0.53	0.21	0.53		
Std. Dev. of Log RT			1324.27**	179.17	1322.26**	179.32		
APOE (0 risk)			5.68	13.31	14.62**	6.72	12.73*	6.31
(1 risk)			8.93*	8.43	9.50**	4.42	6.81	3.99
(2 risk)			0^a^	0^a^	0^a^	0^a^	0^a^	0^a^
COMT (0 risk)			−0.35	11.47	−2.33	6.00	−2.71	5.40
(1 risk)			14.00**	8.39	10.79**	4.46	10.62**	3.93
(2 risk)			0^a^	0^a^	0^a^	0^a^	0^a^	0^a^
DAT1 (0 risk)			33.59**	18.18	20.90**	9.38	17.75*	8.54
(1 risk)			−1.81	7.21	−3.86*	3.71	0.31	3.41
(2 risk)			0^a^	0^a^	0^a^	0^a^	0^a^	0^a^
DBH (0 risk)			3.72	10.15	4.26	5.31	6.76	4.78
(1 risk)			−10.45**	8.11	−7.13**	4.17	−6.41	3.82
(2 risk)			0^a^	0^a^	0^a^	0^a^	0^a^	0^a^
DRD4 (0 risk)			6.17	9.59	2.86	4.97	5.39	4.54
(1 risk)			15.53**	8.36	10.90**	4.27	17.08**	3.96
(2 risk)			0^a^	0^a^	0^a^	0^a^	0^a^	0^a^
APOE (0 risk)*Trial			NS	NS			NS	NS
COMT (0 risk)*Trial			NS	NS			NS	NS
DAT1 (0 risk)*Trial			NS	NS			NS	NS
DBH (0 risk)*Trial			NS	NS			NS	NS
DRD4 (0 risk)*Trial			NS	NS			NS	NS
Covariance parameters								
Repeated measures			5982.33**	0.16	5992.84**	0.16	7201.47**	0.20
Number of parameters	2		31		23		26	
Schwarz’s Bayesian criterion (BIC)	−6286.49		−5057.25		−5216.91		−4705.49	

Significant values for ethnicities indicate that the ethnic group is different from Caucasians on RT values. Other significant values indicate that the estimate is different from zero. We report in the “Results” section tests of fixed effects for gene by trial interactions to determine if slopes differ by genotype

Note that this table shows model parameter estimates when analyzing log transformed RTs. The values have been retransformed. However, to get a rough idea of slope differences by genotype group, please see Table 12 for raw RTs (ms)

*NS* the slopes were not significantly different from zero and so the row has been collapsed

* Significant at *p* < 0.05

** Significant at *p* < 0.01

^a^This parameter has been set to zero

**Table 5:** Estimates from models of the predictors of log-RT for the neutral bright condition

Parameter	Empty model		Full model		Non-significant interaction removed		Covariates removed	
	Mean	SE	Mean	SE	Mean	SE	Mean	SE
Fixed effects								
Intercept	477.68**	1.65	263.25**	18.44	268.68**	15.02	433.18**	13.97
Level 1 (trial-specific)								
Trial			0.02	0.11	−0.02	0.03	0.02	0.05
Side			−0.56	1.68	−0.57	1.67		
Level 2 (individual)								
Age			1.08**	0.20	1.08**	0.19		
Asian			10.84**	5.14	10.84**	5.13	7.81**	2.34
Black			19.90**	10.10	19.87**	10.09	22.12**	4.70
Hispanic			3.94	5.63	3.93	5.63	9.24**	2.51
White			0^a^	0^a^	0^a^	0^a^	0^a^	0^a^
Error rate			−248.00**	164.59	−248.43**	164.46		
Sleepiness (centered)			0.36	0.53	0.36	0.53		
Std. Dev. of Log RT			1200.33**	179.18	1200.84**	179.03		
APOE (0 risk)			11.42**	13.20	13.56**	6.72	16.87**	6.14
(1 risk)			10.96*	8.36	11.37**	4.42	8.35*	3.88
(2 risk)			0^a^	0^a^	0^a^	0^a^	0^a^	0^a^
COMT (0 risk)			−4.90	11.34	−5.18*	5.96	−7.87	5.24
(1 risk)			10.25**	8.32	9.28**	4.43	6.57	3.82
(2 risk)			0^a^	0^a^	0^a^	0^a^	0^a^	0^a^
DAT1 (0 risk)			32.17	18.08	24.02**	9.38	17.47*	8.33
(1 risk)			3.10	7.15	−0.74	3.70	4.91	3.31
(2 risk)			0^a^	0^a^	0^a^	0^a^	0^a^	0^a^
DBH (0 risk)			0.23	10.09	1.41	5.31	2.57	4.66
(1 risk)			−9.06**	8.04	−7.85**	4.17	−5.61	3.72
(2 risk)			0^a^	0^a^	0^a^	0^a^	0^a^	0^a^
DRD4 (0 risk)			7.66	9.55	2.06	4.96	7.04	4.44
(1 risk)			13.79**	8.28	10.17**	4.27	15.39**	3.85
(2 risk)			0^a^	0^a^	0^a^	0^a^	0^a^	0^a^
APOE (0 risk)*Trial			NS	NS			NS	NS
COMT (0 risk)*Trial			NS	NS			NS	NS
DAT1 (0 risk)*Trial			NS	NS			NS	NS
DBH (0 risk)*Trial			NS	NS			NS	NS
DRD4 (0 risk)*Trial			NS	NS			NS	NS
Covariance parameters								
Repeated measures			6076.64**	0.17	6067.27**	0.17	7047.49**	0.20
Number of parameters	2		31		23		26	
Schwarz’s Bayesian criterion (BIC)	−6331.46		−4980.39		−5146.62		−4702.19	

Significant values for ethnicities indicate that the ethnic group is different from Caucasians on RT values. Other significant values indicate that the estimate is different from zero. We report in the “Results” section tests of fixed effects for gene by trial interactions to determine if slopes differ by genotype

Note that this table shows model parameter estimates when analyzing log transformed RTs. The values have been retransformed. However, to get a rough idea of slope differences by genotype group, please see Table 12 for raw RTs (ms)

*NS* the slopes were not significantly different from zero and so the row has been collapsed

* Significant at *p* < 0.05

** Significant at *p* < 0.01

^a^This parameter has been set to zero

**Table 6:** Estimates from models of the predictors of log-RT for the neutral dim condition

Parameter	Empty model		Full model		Non-significant interaction removed		Covariates removed	
	Mean	SE	Mean	SE	Mean	SE	Mean	SE
Fixed effects								
Intercept	479.66**	1.61	266.83**	17.92	268.31**	14.68	435.62**	13.60
Level 1 (trial-specific)								
Trial			−0.01	0.11	−0.02	0.03	−0.01	0.05
Side			−1.68*	1.63	−1.69*	1.63		
Level 2 (individual)								
Age			1.02**	0.19	1.02**	0.19		
Asian			13.72**	5.02	13.68**	5.02	10.85**	2.29
Black			17.15**	9.88	17.13**	9.89	19.42**	4.61
Hispanic			2.36	5.47	2.36	5.48	7.95**	2.44
White			0^a^	0^a^	0^a^	0^a^	0^a^	0^a^
Error rate			−273.99**	160.57	−272.92**	160.72		
Sleepiness (centered)			0.31	0.51	0.31	0.51		
Std. Dev. of Log RT			1221.08**	174.93	1218.86**	175.10		
APOE (0 risk)			9.67	12.84	12.43**	6.57	15.10*	5.99
(1 risk)			8.24*	8.13	9.07**	4.31	5.64	3.79
(2 risk)			0^a^	0^a^	0^a^	0^a^	0^a^	0^a^
COMT (0 risk)			−9.68*	11.02	−5.01*	5.81	−12.11*	5.11
(1 risk)			7.88*	8.06	10.68**	4.32	4.77	3.71
(2 risk)			0^a^	0^a^	0^a^	0^a^	0^a^	0^a^
DAT1 (0 risk)			40.38**	17.81	28.30**	9.18	24.63**	8.23
(1 risk)			4.50	6.94	−0.36	3.61	6.13	3.22
(2 risk)			0^a^	0^a^	0^a^	0^a^	0^a^	0^a^
DBH (0 risk)			−0.12	9.80	3.04	5.16	2.16	4.55
(1 risk)			−6.38	7.81	−5.49**	4.07	−2.99	3.63
(2 risk)			0^a^	0^a^	0^a^	0^a^	0^a^	0^a^
DRD4 (0 risk)			13.79**	9.26	6.25**	4.83	13.32**	4.32
(1 risk)			13.52**	8.06	11.93**	4.16	15.10**	3.75
(2 risk)			0^a^	0^a^	0^a^	0^a^	0^a^	0^a^
APOE (0 risk)*Trial			NS	NS			NS	NS
COMT (0 risk)*Trial			NS	NS			NS	NS
DAT1 (0 risk)*Trial			NS	NS			NS	NS
DBH (0 risk)*Trial			NS	NS			NS	NS
DRD4 (0 risk)*Trial			−0.08*	0.08			−0.07	0.04
(1 risk)			−0.02	0.07			−0.01	0.03
(2 risk)			0^a^	0^a^			0^a^	0^a^
Covariance parameters								
Repeated measures			5751.24**	0.16	5763.32**	0.16	6703.64**	0.19
Number of parameters	2		31		23		26	
Schwarz’s Bayesian criterion (BIC)	−6475.39		−5104.74		−5263.70		−4816.56	

Significant values for ethnicities indicate that the ethnic group is different from Caucasians on RT values. Other significant values indicate that the estimate is different from zero. We report in the “Results” section tests of fixed effects for gene by trial interactions to determine if slopes differ by genotype

Note that this table shows model parameter estimates when analyzing log transformed RTs. The values have been retransformed. However, to get a rough idea of slope differences by genotype group, please see Table 12 for raw RTs (ms)

*NS* the slopes were not significantly different from zero and so the row has been collapsed

* Significant at *p* < 0.05

** Significant at *p* < 0.01

^a^This parameter has been set to zero

**Table 7:** Estimates from models of the predictors of log-RT for the no cue condition

Parameter	Empty model		Full model		Non-significant interaction removed		Covariates removed	
	Mean	SE	Mean	SE	Mean	SE	Mean	SE
Fixed effects								
Intercept	534.95**	1.78	300.44**	20.60	300.99**	16.99	487.29**	15.68
Level 1 (trial-specific)								
Trial			0.05	0.12	0.05*	0.04	0.05	0.06
Side			−2.94**	1.86	−2.94**	1.86		
Level 2 (individual)								
Age			0.99**	0.22	0.99**	0.22		
Asian			9.34**	5.73	9.34**	5.72	7.12**	2.60
Black			0.84	11.41	0.81	11.39	2.78	5.30
Hispanic			−1.84	6.23	−1.85	6.23	5.52*	2.77
White			0^a^	0^a^	0^a^	0^a^	0^a^	0^a^
Error rate			−164.14*	182.85	−164.14*	182.69		
Sleepiness (centered)			0.78**	0.59	0.78**	0.59		
Std. Dev. of Log RT			1333.41**	198.90	1333.80**	198.75		
APOE (0 risk)			−2.28	14.85	7.14*	7.47	2.35	6.89
(1 risk)			8.54*	9.41	8.93**	4.92	5.09	4.37
(2 risk)			0^a^	0^a^	0^a^	0^a^	0^a^	0^a^
COMT (0 risk)			−0.99	12.74	−2.60	6.61	−5.46	5.88
(1 risk)			10.04*	9.33	9.84**	4.92	5.13	4.29
(2 risk)			0^a^	0^a^	0^a^	0^a^	0^a^	0^a^
DAT1 (0 risk)			39.93**	20.25	39.95**	18.92	22.73*	9.32
(1 risk)			10.96**	8.04	11.53**	7.95	12.19**	3.72
(2 risk)			0^a^	0^a^	0^a^	0^a^	0^a^	0^a^
DBH (0 risk)			4.27	11.39	3.09	5.91	4.77	5.26
(1 risk)			−4.79	9.06	−4.42*	4.65	−1.47	4.18
(2 risk)			0^a^	0^a^	0^a^	0^a^	0^a^	0^a^
DRD4 (0 risk)			6.72	10.73	4.36	5.51	7.22	4.98
(1 risk)			9.36*	9.31	7.54**	4.74	11.38**	4.32
(2 risk)			0^a^	0^a^	0^a^	0^a^	0^a^	0^a^
APOE (0 risk)*Trial			NS	NS			NS	NS
COMT (0 risk)*Trial			NS	NS			NS	NS
DAT1 (0 risk)*Trial			−0.19*	0.17	−0.19**	0.16	−0.19*	0.08
(1 risk)			−0.08**	0.07	−0.09**	0.07	−0.08**	0.03
(2 risk)			0^a^	0^a^	0^a^	0^a^	0^a^	0^a^
DBH (0 risk)*Trial			NS	NS			NS	NS
DRD4 (0 risk)*Trial			NS	NS			NS	NS
Covariance parameters								
Repeated measures			7500.47**	0.19	7488.63**	0.19	8649.11**	0.21
Number of parameters	2		31		23		26	
Schwarz’s Bayesian criterion (BIC)	−6532.05		−4998.91		−5132.96		−4733.59	

Significant values for ethnicities indicate that the ethnic group is different from Caucasians on RT values. Other significant values indicate that the estimate is different from zero. We report in the “Results” section tests of fixed effects for gene by trial interactions to determine if slopes differ by genotype

Note that this table shows model parameter estimates when analyzing log transformed RTs. The values have been retransformed. However, to get a rough idea of slope differences by genotype group, please see Table 12 for raw RTs (ms)

*NS* the slopes were not significantly different from zero and so the row has been collapsed

* Significant at *p* < 0.05

** Significant at *p* < 0.01

^a^This parameter has been set to zero

**Table 8:** Estimates from models of the predictors of log-RT for the single bright valid condition

Parameter	Empty model		Full model		Non-significant interaction removed		Covariates removed	
	Mean	SE	Mean	SE	Mean	SE	Mean	SE
Fixed effects								
Intercept	461.40**	1.85	269.02**	21.48	263.75**	17.88	428.23**	15.85
Level 1 (trial-specific)								
Trial			−0.03	0.13	0.01	0.04	−0.03	0.06
Side			−3.23**	1.96	−3.23**	1.96		
Level 2 (individual)								
Age			1.20**	0.23	1.20**	0.23		
Asian			10.27**	6.04	10.24**	6.04	5.68*	2.69
Black			19.23**	11.84	19.23**	11.83	20.58**	5.38
Hispanic			3.73	6.58	3.73	6.58	7.84**	2.86
White			0^a^	0^a^	0^a^	0^a^	0^a^	0^a^
Error rate			−295.29**	191.84	−295.91**	191.74		
Sleepiness (centered)			0.50	0.62	0.50	0.62		
Std. Dev. of Log RT			1017.83**	209.69	1017.63**	209.58		
APOE (0 risk)			5.10	15.26	8.50*	7.89	10.72	6.93
(1 risk)			7.70	9.65	10.97**	5.18	5.86	4.38
(2 risk)			0^a^	0^a^	0^a^	0^a^	0^a^	0^a^
COMT (0 risk)			−9.88	13.09	−7.11*	6.98	−11.93*	5.91
(1 risk)			13.59**	9.60	11.42**	5.19	10.59*	4.30
(2 risk)			0^a^	0^a^	0^a^	0^a^	0^a^	0^a^
DAT1 (0 risk)			41.25**	20.95	42.17	19.66**	26.69**	9.42
(1 risk)			5.24	8.27	5.73	8.17	6.49	3.74
(2 risk)			0^a^	0^a^	0^a^	0^a^	0^a^	0^a^
DBH (0 risk)			−2.28	11.63	2.24	6.21	1.10	5.25
(1 risk)			−11.52**	9.30	−8.05**	4.89	−7.46	4.20
(2 risk)			0^a^	0^a^	0^a^	0^a^	0^a^	0^a^
DRD4 (0 risk)			1.96	11.02	−0.81	5.81	0.77	5.00
(1 risk)			10.18*	9.57	11.14**	5.00	10.96*	4.34
(2 risk)			0^a^	0^a^	0^a^	0^a^	0^a^	0^a^
APOE (0 risk)*Trial			NS	NS			NS	NS
COMT (0 risk)*Trial			NS	NS			NS	NS
DAT1 (0 risk)*Trial			−0.16*	0.18	−0.17**	0.16	−0.16*	0.08
(1 risk)			−0.05	0.07	−0.05	0.07	−0.05	0.03
(2 risk)			0^a^	0^a^	0^a^	0^a^	0^a^	0^a^
DBH (0 risk)*Trial			NS	NS			NS	NS
DRD4 (0 risk)*Trial			NS	NS			NS	NS
Covariance parameters								
Repeated measures			8348.67**	0.24	8340.00**	0.24	9330.38**	0.27
Number of parameters	2		31		23		26	
Schwarz’s Bayesian criterion (BIC)	−5503.84		−4716.22		−4306.30		−3975.20	

Significant values for ethnicities indicate that the ethnic group is different from Caucasians on RT values. Other significant values indicate that the estimate is different from zero. We report in the “Results” section tests of fixed effects for gene by trial interactions to determine if slopes differ by genotype

Note that this table shows model parameter estimates when analyzing log transformed RTs. The values have been retransformed. However, to get a rough idea of slope differences by genotype group, please see Table 12 for raw RTs (ms)

*NS* the slopes were not significantly different from zero and so the row has been collapsed

* Significant at *p* < 0.05

** Significant at *p* < 0.01

^a^This parameter has been set to zero

**Table 9:** Estimates from models of the predictors of log-RT for the single bright invalid condition

Parameter	Empty model		Full model		Non-significant interaction removed		Covariates removed	
	Mean	SE	Mean	SE	Mean	SE	Mean	SE
Fixed effects								
Intercept	512.11**	1.87	283.91**	20.41	274.15**	16.76	486.91**	15.61
Level 1 (trial-specific)								
Trial			−0.09	0.12	0.00	0.00	−0.08	0.06
Side			−3.81**	1.87	−3.83**	1.87		
Level 2 (individual)								
Age			1.25**	0.22	1.26**	0.03		
Asian			16.91**	5.74	16.86**	5.73	14.14**	2.64
Black			20.64**	11.44	20.53**	11.45	23.58**	5.38
Hispanic			9.13**	6.30	9.34**	6.19	14.65**	2.84
White			0^a^	0^a^	0^a^	0^a^	0^a^	0^a^
Error rate			−272.10**	184.83	−273.22**	185.21		
Sleepiness (centered)			0.11	0.59	0.12	0.22		
Std. Dev. of Log RT			1415.86**	200.67	1417.82**	200.84		
APOE (0 risk)			−1.39	14.60	9.08**	7.45	4.54	6.87
(1 risk)			3.09	9.28	8.67**	4.93	−0.21	4.36
(2 risk)			0^a^	0^a^	0^a^	0^a^	0^a^	0^a^
COMT (0 risk)			−7.05	12.53	−4.18	6.51	−9.05	5.86
(1 risk)			3.72	9.19	10.57**	4.75	0.85	4.27
(2 risk)			0^a^	0^a^	0^a^	0^a^	0^a^	0^a^
DAT1 (0 risk)			28.01**	20.01	21.05**	10.35	11.01	9.33
(1 risk)			−0.72	7.92	−6.07**	4.06	1.57	3.71
(2 risk)			0^a^	0^a^	0^a^	0^a^	0^a^	0^a^
DBH (0 risk)			−4.41	11.12	−0.95	5.86	−1.01	5.20
(1 risk)			−10.45**	8.92	−6.89**	4.66	−5.97	4.18
(2 risk)			0^a^	0^a^	0^a^	0^a^	0^a^	0^a^
DRD4 (0 risk)			9.13*	10.58	5.60*	5.54	8.57	4.97
(1 risk)			12.12**	9.16	11.21**	4.75	13.52**	4.31
(2 risk)			0^a^	0^a^	0^a^	0^a^	0^a^	0^a^
APOE (0 risk)*Trial			NS	NS			NS	NS
COMT (0 risk)*Trial			0.03	0.11			0.02	0.05
(1 risk)			0.07*	0.08			0.07	0.04
(2 risk)			0^a^	0^a^			0^a^	0^a^
DAT1 (0 risk)*Trial			NS	NS			NS	NS
DBH (0 risk)*Trial			NS	NS			NS	NS
DRD4 (0 risk)*Trial			NS	NS			NS	NS
Covariance parameters								
Repeated measures			7308.26**	0.19	7339.45**	0.19	8701.69**	0.23
Number of parameters	2		31		23		26	
Schwarz’s Bayesian criterion (BIC)	−5900.16		−4716.22		−4881.78		−4393.91	

Significant values for ethnicities indicate that the ethnic group is different from Caucasians on RT values. Other significant values indicate that the estimate is different from zero. We report in the “Results” section tests of fixed effects for gene by trial interactions to determine if slopes differ by genotype

Note that this table shows model parameter estimates when analyzing log transformed RTs. The values have been retransformed. However, to get a rough idea of slope differences by genotype group, please see Table 12 for raw RTs (ms)

*NS* the slopes were not significantly different from zero and so the row has been collapsed

* Significant at *p* < 0.05

** Significant at *p* < 0.01

^a^This parameter has been set to zero

**Table 10:** Estimates from models of the predictors of log-RT for the single dim valid condition

Parameter	Empty model		Full model		Non-significant interaction removed		Covariates removed	
	Mean	SE	Mean	SE	Mean	SE	Mean	SE
Fixed effects								
Intercept	477.28**	1.91	293.25**	22.29	273.05**	18.43	493.33**	16.64
Level 1 (trial-specific)								
Trial			−0.19**	0.13	−0.04*	0.05	−0.19**	0.06
Side			−0.61	2.02	−0.61	2.02		
Level 2 (individual)								
Age			1.17**	0.24	1.17**	0.24		
Asian			8.72**	6.19	8.71**	6.20	4.84	2.76
Black			12.48*	12.25	12.45	12.25	14.13*	5.58
Hispanic			0.68	6.80	0.71	6.80	6.62*	2.97
White			0^a^	0^a^	0^a^	0^a^	0^a^	0^a^
Error rate			−252.03**	197.47	−251.97**	197.60		
Sleepiness (centered)			0.72**	0.64	0.73**	0.64		
Std. Dev. of Log RT			1223.84**	215.62	1224.63**	215.76		
APOE (0 risk)			6.57	15.98	12.47**	8.11	12.08	7.27
(1 risk)			1.92	10.10	9.10**	5.33	−0.80	4.59
(2 risk)			0^a^	0^a^	0^a^	0^a^	0^a^	0^a^
COMT (0 risk)			−14.77*	13.69	−8.43**	7.19	−18.57**	6.19
(1 risk)			2.49	10.06	7.65**	5.36	−1.92	4.52
(2 risk)			0^a^	0^a^	0^a^	0^a^	0^a^	0^a^
DAT1 (0 risk)			37.45**	21.89	46.02**	20.55	20.64*	9.88
(1 risk)			8.89*	8.65	10.10**	8.57	10.28**	3.92
(2 risk)			0^a^	0^a^	0^a^	0^a^	0^a^	0^a^
DBH (0 risk)			−5.28	12.17	2.22	6.40	−3.16	5.51
(1 risk)			−10.52	9.73	−8.29**	5.04	−6.73	4.41
(2 risk)			0^a^	0^a^	0^a^	0^a^	0^a^	0^a^
DRD4 (0 risk)			0.23	11.50	1.10	5.99	−0.28	5.23
(1 risk)			3.37	10.02	8.39**	5.16	4.66	4.55
(2 risk)			0^a^	0^a^	0^a^	0^a^	0^a^	0^a^
APOE (0 risk)*Trial			NS	NS			NS	NS
COMT (0 risk)*Trial			NS	NS			NS	NS
DAT1 (0 risk)*Trial			−0.15	0.19	−0.23**	0.17	−0.15	0.09
(1 risk)			−0.08**	0.07	−0.10**	0.07	−0.08*	0.03
(2 risk)			0^a^	0^a^	0^a^	0^a^	0^a^	0^a^
DBH (0 risk)*Trial			NS	NS			NS	NS
DRD4 (0 risk)*Trial			NS	NS			NS	NS
Covariance parameters								
Repeated measures			8836.79**	0.24	8848.44**	0.24	9931.41**	0.27
Number of parameters	2		31		23		26	
Schwarz’s Bayesian criterion (BIC)	−5496.49		−4197.00		−4322.01		−3984.53	

Significant values for ethnicities indicate that the ethnic group is different from Caucasians on RT values. Other significant values indicate that the estimate is different from zero. We report in the “Results” section tests of fixed effects for gene by trial interactions to determine if slopes differ by genotype

Note that this table shows model parameter estimates when analyzing log transformed RTs. The values have been retransformed. However, to get a rough idea of slope differences by genotype group, please see Table 12 for raw RTs (ms)

*NS* the slopes were not significantly different from zero and so the row has been collapsed

* Significant at *p* < 0.05

** Significant at *p* < 0.01

^a^This parameter has been set to zero

**Table 11:** Estimates from models of the predictors of log-RT for the single dim invalid condition

Parameter	Empty model		Full model		Non-significant interaction removed		Covariates removed	
	Mean	SE	Mean	SE	Mean	SE	Mean	SE
Fixed effects								
Intercept	504.98**	1.79	270.49**	19.91	265.01**	16.37	476.80**	15.59
Level 1 (trial-specific)								
Trial			−0.02	0.12	0.03	0.04	−0.01	0.06
Side			−2.11**	1.79	−2.11**	1.79		
Level 2 (individual)								
Age			1.17**	0.21	1.17**	0.21		
Asian			12.63**	5.49	12.62**	5.49	10.09**	2.54
Black			23.52**	10.78	23.52**	10.78	26.58**	5.11
Hispanic			7.29**	6.03	7.30**	6.03	13.76**	2.74
White			0^a^	0^a^	0^a^	0^a^	0^a^	0^a^
Error rate			−295.66**	176.72	−295.90**	176.67		
Sleepiness (centered)			0.09	0.56	0.10	0.56		
Std. Dev. of Log RT			1517.02**	191.91	1516.95**	191.84		
APOE (0 risk)			5.25	14.55	9.34*	7.18	11.91	6.89
(1 risk)			9.71*	9.19	9.15**	4.73	6.47	4.35
(2 risk)			0^a^	0^a^	0^a^	0^a^	0^a^	0^a^
COMT (0 risk)			−11.84*	12.37	−6.78**	6.39	−14.87*	5.83
(1 risk)			6.42	9.06	6.74**	4.73	2.67	4.25
(2 risk)			0^a^	0^a^	0^a^	0^a^	0^a^	0^a^
DAT1 (0 risk)			39.22**	19.59	39.02**	18.26	22.48*	9.21
(1 risk)			−2.49	7.83	−2.03	7.73	−0.18	3.70
(2 risk)			0^a^	0^a^	0^a^	0^a^	0^a^	0^a^
DBH (0 risk)			−3.39	11.02	0.67	5.69	−0.04	5.19
(1 risk)			−14.60**	8.80	−8.20**	4.47	−10.61**	4.15
(2 risk)			0^a^	0^a^	0^a^	0^a^	0^a^	0^a^
DRD4 (0 risk)			4.87	10.43	2.29	5.32	5.29	4.94
(1 risk)			11.66**	9.06	11.82**	4.57	13.75**	4.29
(2 risk)			0^a^	0^a^	0^a^	0^a^	0^a^	0^a^
APOE (0 risk)*Trial			NS	NS			NS	NS
COMT (0 risk)*Trial			NS	NS			NS	NS
DAT1 (0 risk)*Trial			−0.19**	0.17	−0.19**	0.15	−0.19*	0.08
(1 risk)			−0.06	0.07	−0.06*	0.07	−0.06	0.03
(2 risk)			0^a^	0^a^	0^a^	0^a^	0^a^	0^a^
DBH (0 risk)*Trial			NS	NS			NS	NS
DRD4 (0 risk)*Trial			NS	NS			NS	NS
Covariance parameters								
Repeated measures			6817.78**	0.18	6814.26**	0.18	8216.45**	0.22
Number of parameters	2		31		23		26	
Schwarz’s Bayesian criterion (BIC)	−6108.62		−4872.73		−5003.79		−4520.07	

Significant values for ethnicities indicate that the ethnic group is different from Caucasians on RT values. Other significant values indicate that the estimate is different from zero. We report in the “Results” section tests of fixed effects for gene by trial interactions to determine if slopes differ by genotype

Note that this table shows model parameter estimates when analyzing log transformed RTs. The values have been retransformed. However, to get a rough idea of slope differences by genotype group, please see Table 12 for raw RTs (ms)

*NS* the slopes were not significantly different from zero and so the row has been collapsed

* Significant at *p* < 0.05

** Significant at *p* < 0.01

^a^This parameter has been set to zero

**Table 12:** Slopes from the regression of the untransformed RT (msec) on trial

	Dual by bright	Dual by dim	Neutral both bright	Neutral both dim	No cue	Single bright valid	Single bright invalid	Single dim valid	Single dim invalid
*APOE* rs429358 and rs7412									
ε2/ε3	−0.005	0.091	0.091	0.081	0.242	0.072	0.225	−0.132	0.140
ε3/ε3	−0.108	−0.168	−0.062	−0.047	−0.011	−0.006	−0.012	−0.161	−0.029
Any ε4	−0.142	−0.183	−0.083	−0.112	−0.061	−0.099	−0.164	−0.339	−0.069
*COMT* rs4680									
A/A	−0.050	−0.150	−0.059	−0.024	−0.090	−0.022	−0.102	−0.202	0.043
A/G	−0.079	−0.145	−0.044	−0.035	0.021	−0.046	0.040	−0.171	0.007
G/G	−0.177	−0.166	−0.068	−0.110	−0.022	−0.025	−0.159	−0.296	−0.080
*DAT1* intron 8 VNTR									
5R/5R	−0.192	−0.362	−0.154	−0.277	*−0.267*	−0.385	−0.409	*−0.559*	*−0.435*
5R/6R	−0.192	−0.211	−0.116	−0.153	*−0.129*	−0.143	−0.123	*−0.360*	*−0.098*
6R/6R	−0.031	−0.105	−0.008	0.023	*0.083*	0.035	0.043	*−0.093*	*0.083*
*DBH* rs1108580									
G/G	−0.149	−0.135	−0.074	−0.053	−0.053	0.043	−0.030	−0.131	−0.027
G/A	−0.066	−0.098	−0.022	−0.017	0.025	−0.017	0.008	−0.212	0.064
A/A	−0.139	−0.239	−0.090	−0.107	−0.032	−0.124	−0.122	−0.285	−0.154
*DRD4* rs747302									
G/G	−0.224	−0.188	−0.115	−0.177	−0.046	−0.107	−0.102	−0.242	−0.054
G/C	−0.113	−0.215	−0.117	−0.054	−0.026	−0.037	−0.048	−0.169	−0.021
C/C	−0.002	−0.042	0.029	0.030	0.057	0.001	0.027	−0.275	0.023

For ease of interpretation, this table presents slopes of untransformed RT rather than log transformed RT. Italic values indicate significant RT slope differences across genotype by primary measure. To calculate the increase or decrease in average RT across 200 trials for individuals with a given genotype, simply multiply the slopes in the table by 200 trials. For example, for the DAT1 5R/5R genotype for the Single Dim Valid condition: mean(RT change) = 200 trials × (−0.56) msec/trial = −112 ms across 200 trials (subjects with this genotype responded more than 100 ms faster on trials at the end of the experiment than at the beginning)

Three of the reduced (final) models had slopes that were significant. In these cases it was the *DAT1* by trial interactions that were significant. The model with slopes and without covariates for *No Cue*^3^ was significantly better than the model with all predictors (the full model) at χ^2^ (5, N = 2559) = 265.32, *p* < 0.001. The *DAT1* × Trial interaction (the RT slope) for *No Cue* was the only significant slope, *F*(2, 2137) = 5.15, *p* = 0.01. There were also main effects for *DAT1* (*F*[2, 2137] = 6.93, *p* = 0.001) and *DRD4* (*F*[2, 2137] = 3.48, *p* = 0.03), but not for Trial (*p* = 0.36). The zero risk allele group (who had no DAT1 intron 8 alleles associated with ADHD) had slopes that were significantly steeper (averaging 0.11 ms more of a decrease in RT per trial) than the one risk allele group, which in turn averaged 0.08 ms more of a decrease in RT per trial than the two risk allele group. The two-risk allele group had an estimate set to zero in the multilevel model (see Table 7). The zero-risk allele group was significantly different from a flat slope, (*t*[2137] = −2.40, *p* = 0.02), as was the one-risk allele group (*t*[2137] = −2.60, *p* = 0.01). The differences are easier to visualize in Table 12 where untransformed RT values show that subjects with zero risk alleles (6R/6R) got about 53 ms faster over the course of the task, while those with two risk alleles (5R/5R) slowed down across the course of the experiment by approximately 17 ms.

The final model for *Single Dim Valid* was significantly better than the model with all predictors at χ^2^ (5, N = 2559) = 212.48, *p* < 0.001. The *DAT1* × Trial interaction was significant at *F*(2, 2139) = 3.89, *p* = 0.02. The main effects were significant for *DAT1* (*F*[2, 2139] = 4.65, *p* = 0.01), *COMT* (*F*[2, 2139] = 5.35, *p* = 0.01), and Trial (*F*[1, 2139] = 15.91, *p* < 0.001). The zero risk group had slopes that were significantly steeper (averaging 0.07 ms faster per trial; see Table 10) than individuals in the one risk allele group (who averaged 0.08 ms faster per trial than those in the two risk allele group). Those in the zero risk allele group had slopes that were marginally different from a flat slope, *t*(2139) = −1.75, *p* = 0.08. Those in the one-risk allele group had slopes that were significantly different from a flat slope, *t*(2139) = −2.50, *p* = 0.01. As can be seen in Table 12, subjects with zero risk alleles responded approximately 112 ms faster over the course of the task, while those with two risk alleles (5R/5R) were responding only 19 ms faster by the end of the task.

The final model for *Single Dim Invalid* was significantly better than the model with all predictors at χ^2^ (5, N = 2513) = 352.66, p < 0.001. The main effects were significant for *DAT1* (*F*[2, 2099] = 3.18, *p* = 0.04), *COMT* (*F*[2, 2099] = 5.78, *p* = 0.003), *DBH* (*F*[2, 2099] = 4.14, *p* = 0.02), and *DRD4* (*F*[2, 2099] = 5.34, *p* = 0.01). The main effect for Trial was not significant (*p* = 0.20).The *DAT1* zero risk allele group has slopes that are significantly larger (averaging a decrease of 0.13 ms more per trial than individuals in the one risk allele group (who have slopes that decrease 0.06 ms per trial faster than the two risk allele group). The zero risk allele group had slopes that were significantly different from zero (*t*[2099] = −2.36, *p* = 0.02), but the one risk allele group was only marginally different from a flat slope, *t*(2099) = −1.75, *p* = 0.08. Subjects with zero risk alleles got about 87 ms faster over the course of the task, while those with two risk alleles (5R/5R) were responding slower by about 17 ms by the end of the task.

## Discussion

One can think of variability in RT in an attention task across repeated trials in two ways: (1) random variability from trial to trial that fluctuates around some mean RT, and (2) a systematic RT slope across trials that tends to make latter RTs slower (or faster) on average than earlier RTs. Because it is common to study the moment-to-moment variability of behavioral or physiological measures between genotypes, it is especially interesting to find that genotype also predicts the latter kind of variability in RT in a cued orienting task: the linear component of changes across trials in RT (slope RT). This was true even after covarying sleepiness, age, self-reported ethnicity, error rate, and moment-to-moment variability, although the statistical analysis led us to settle on the simpler models without these covariates. *DAT1* was the only gene in the simpler models that interacted with trial number in predicting RT slopes. *DAT1* produces a protein involved in the transport of dopamine from the synapse. We note that our task produced the typical costs and benefits of a reflexive attention task [1, 36]. Therefore, our finding is intriguing because it implies that it is not the nature of the task per se (i.e., whether it is designed to involve effortful/sustained attention or not) but possibly that there is a task that transpires across an extended time period.

For *DAT1*, the mean slope increased (subjects got slower) across genotypes with increasing numbers of risk alleles for three outcome measures that were significant. The significant outcome measures were conditions in which there was no cue and in which the single cues were dim (both valid and invalid). The conditions that were not significantly associated with *DAT1* are also interesting. These include the *Dual Asymmetric Bright* (the target appeared near the brighter of two simultaneous cues) and *Dual Asymmetric Dim* conditions (the target appeared near the dimmer of two simultaneous cues), and the *Neutral Both Bright* and *Neutral Both Dim* conditions (the target appeared near either of two identical cues). The *Single Bright Valid* and *Invalid* cues were also not significant. This all suggests that it is not validity that distinguishes genotype groups (because subjects with two risk alleles do not respond more quickly across trials with either valid or invalid pre-cues). It may be, however, that cue luminance matters (because those with two risk alleles do not respond more quickly across trials with single, dim cues) and cue number matters (because dual cues did not distinguish between subjects in the different genotype groups). The risk allele for our genetic marker on *DAT1* (six repeats of a VNTR) increases production of the dopamine transporter so that the dopaminergic signal is often terminated too soon [37].

One possible conclusion regarding genetic influence on the no-cue and single, dim cue conditions is that bright cues are too salient to distinguish between genotype groups (i.e., all groups performed at ceiling) because dopamine variability in the general population is sufficient for this performance. Another possible conclusion regarding genetic influence is that the addition of a second cue induces subjects to perform more similarly to each other across trials than a single cue, again, because dopamine is sufficient with two cues. In a followup analysis with *DAT1,* the number of cues showed a slight statistical trend for association with RT slope (*p* = 0.15) with steeper improvements in RT across trials for the no risk allele (5R/5R) group with one cue trials than with two cue trials. This finding is worth investigation in a future study because it suggests the possibility of an effect in which RT slopes across trials are approximately equal between genotype groups when there are more cues (perhaps because attention is divided) and if they have any copy of the risk allele (6R). Conversely, they respond more quickly across trials if there is only one cue and they have no risk alleles. In other words, subjects respond more similarly across the course of the task when there are two cues, but single cues reflexively capture attention and require a disengage step, which may become more effortful for some subjects over the course of the task.

Why would the *DAT1* genotypes be associated with systematic *differences* in the rate of responding across trials? There are three possible outcomes when examining the interaction between gene and trial in predicting logarithm-transformed RT: (1) subjects can respond more quickly across trials (negative RT slope as trial number increases), (2) subjects can respond equally quickly across trials, or (3) subjects can respond more slowly across trials (positive RT slope as trial number increases). Also keep in mind that a significant difference in RT slope across trials between genotype groups does not always entail a negative RT slope in one group and a positive RT slope in another group. It could simply mean that the RT slope was less negative in one group than in another, while both groups could show negative slopes. In all cases, the significant effects consisted of less negative or more positive RT slopes across trials associated with more risk alleles for the various genes (as defined by association with ADHD). Recall that possession of more copies of the risk allele on *DAT1* would tend to result in faster transport of dopamine from the synapses. The nature of the behavioral effect is that subjects tend to slow down across the course of the experiment, especially with the 5R/6R and 6R/6R genotypes. One possible explanation for this effect, then, is that an optimal level of dopamine is required to maintain alertness [22, 28, 38–42]. This is actually not different from saying that the association between *DAT1* and RT slope may be related to vigilance or sustained attention, even though this is a reflexive attention task. There is a large literature for genetic effects of dopamine related genes for sustained attention [22, 28, 41, 42].

In the case of the current results with the reflexive attention task, waning vigilance across the course of the experiment would tend to lead to gradually longer target detection times and longer RTs on average despite the reflexive nature of the cue-target trials. This hypothesis would, of course, predict that possession of more *DAT1* risk alleles should have caused a general slowing across trials in all conditions. Instead, this effect was observed in three of nine conditions. It is possible that issues of power prevented detection of the effect in the other conditions. Nevertheless, dopamine availability, cue luminance and cue number could all be important. We have provided evidence that there are genetic influences on RT trends over the course of a reflexive attention task and not simply on terms of moment-to-moment RT variability.

Of course, chance is another possibility that could explain our results. In fact, the biggest limitation of this study regards sample size which all other things being equal tends to make results less replicable. While some authors caution against using sample sizes smaller than 500 to explore interaction effects [43], others have noted sufficient power and stability to explore cross-level interactions in multilevel models with sample sizes of 30–50 level-2 units (individual subjects in this study) [44, 45] and at least 30 level-1 units (the trials in this study) per level-2 unit. Our study certainly met the latter criterion. The best way to handle this question is to replicate this study.

Another limitation could be the heterogeneity of our sample. While we see advantages to including multiple ethnicities and ages, population stratification as an artifact is certainly more likely as the heterogeneity of the sample increases. This would argue for a replication with a larger overall sample and more balanced ethnic subsamples. This could lead to more certainty in the generalizability of the results. It would also be advantageous to explore associations with more genetic markers including those that are associated with neurotransmitters other than dopamine.

## Conclusions

Over the course of a reflexive visual attention experiment with repeated trials of the same conditions, some subjects fail to respond more quickly to identical stimuli, but others respond more quickly as the task progresses. This is interesting because it suggests the possibility of similar biological mechanisms for sustaining attention to exogenous and endogenous cues [9, 46, 47]. However, there are other possibilities. Responding more slowly to later presentations of the same stimulus could indicate, for example, fatigue, a build-up of trial-to-trial inhibition, or greater interference from immediately preceding trials. Responding more quickly to the same stimulus in later presentations could be considered a simple form of learning. The subject’s task was simply to make a choice response (right versus left) to a particular target that appeared across trials either unaccompanied by a temporally preceding cue, or accompanied by such a cue that could appear spatially near the target’s position or contralaterally across the visual field from the location of the target.

We asked whether individual differences in these RT slopes in response to the cue-target manipulation were associated with genetic markers for five genes: *COMT*, *DAT1*, *DBH*, *DRD4*, and *APOE.* These genes were chosen because they have been shown to be related to various aspects of visual attention in previous research, and because in some cases, there is a plausible biological pathway from the gene to the phenotypes that we used to study visual orienting. *DAT1* genotype was associated with variations in these slopes in three different cue-target conditions. The mean slope increased with increasing numbers of risk alleles. A larger positive mean slope implies that the difference in RT between early trials with a given stimulus and later trials with that same stimulus was greater (greater RT slowing) for subjects carrying more of the *DAT1* risk alleles. The risk allele was determined based primarily on previous research on related sustained attention tasks.

Previous work, including our own, has shown associations between the mean RT in different conditions and various genes, but such associations are like snapshots of genotype/phenotype relations in the sense that the phenotype is not extended in time. It reflects a static aspect of visual attention. In contrast, the phenotypes that we examined were by definition extended in time because they were defined by the slope of RT across repeated presentations of the same stimulus. It was primarily *DAT1* that was associated with these temporally extended phenotypes. The phenotypes reflect how speeded attentional choices change over perhaps a 30 min period filled with repetitions of multiple trial types. However, this temporally extended phenotype could reflect the ability to maintain attentional arousal—the ability to learn to recognize the spatiotemporal configurations of the various trial types in order to respond more discriminatively when they appear again—or other processes that systematically alter RTs across an extended period of time. Our results suggest that the *DAT1* gene is a promising candidate for understanding the underlying pathways involved in these more temporally extended aspects of either reflexive or sustained visual attention.

## Methods

### Subjects and data set

This study was approved by Rice University’s Internal Review Board for Human Subjects in Research and conducted in accordance with ethical guidelines established by the Office for Human Research Protections at the US Department of Health and Human Services. We tested subjects between the ages of 18 and 61 years old from the general population. Most of the subjects (*n* = 107) were Rice University students, and the remaining subjects (*n* = 54) were recruited from the surrounding community by posting to neighborhood email groups (see Table 13 for a comparison of these two recruiting sources and Table 14 for demographics). Prior to completing the visual orienting task, subjects signed a consent form and completed an intake questionnaire that included questions on basic demographics, tobacco use, sleepiness (using the Epworth Sleepiness Scale) [48], and attentional disorders affecting the subject and their biological relatives. Subjects provided a saliva sample from which we extracted DNA to obtain information on genotypes for five genetic markers (see Table 1).

**Table 13:** Comparison of these two recruiting sources

	T	df	Sig. (2-tailed)	Mean difference	Std. error difference
Age	−9.89	54.95	0.00	−14.89	1.51
Gender	−0.34	145	0.73	−0.03	0.09
APOE	0.78	133	0.44	0.08	0.10
COMT	0.31	143	0.76	0.03	0.11
DAT1	−0.30	138	0.77	−0.03	0.10
DBH	1.50	142	0.14	0.19	0.12
DRD4	−2.31	132	0.02	−0.32	0.14

The two recruiting sources were Rice University students and subjects recruited from a suburb of Houston. As can be seen, the subjects from the two groups differed on age (the suburb sample was older) and *DRD4* genotype. Multilevel modeling accounts for the fact that trials are not independent (certain trials come from certain individuals) and so these differences are less of a concern than they might be with other statistical analyses

**Table 14:** Covariates by recruiting source

	SD of log RT	Log RT	Age	Sleepiness	Error rate	Ethnicity			
	Mean	Mean	Mean	Mean	Mean	Asian Count	Black Count	Hispanic Count	Caucasian Count
Rice									
Male	2.69	0.07	20	8	0.02	12	2	7	22
Female	2.68	0.07	20	9	0.02	12	4	10	26
Community									
Male	2.67	0.06	36	9	0.02	0	1	1	20
Female	2.69	0.06	35	9	0.02	0	0	0	30

As can be seen, the primary demographic differences between the two recruiting sources are greater age and less ethnic diversity in the community sample. The community sample was recruited to increase age range. Reduced ethnic diversity is a common byproduct of recruiting in American suburban areas

A subject’s data were removed from the data set if he or she had error rates over 10 % on the behavioral task, could not be classified into a single ethnicity, or reported a history of a serious neurological disorder. Of the 161 subjects, eight were excluded for having an error rate over 10 %. This decision was based on the distribution of error rates (including catch trial errors). High error-rates could indicate that subjects might not be motivated or understand the task. The error rate for the remaining subjects averaged 2 % (SD = 0.02). One subject was excluded for not being classifiable to a single ethnicity. However, eleven individuals with dual ethnicity were classifiable based on the pattern of their genes. We calculated the proportion of each single ethnic group having each genotype. Then we looked at the genotypes of those individuals reporting two ethnicities. If there was a pattern (e.g., if a person’s genotype matched the most common genotype for Hispanics on 4 out of 5 genes) and one of the two ethnicities they reported was Hispanic, then they were coded as Hispanic. We compared the results to the best distinguishing genotype for each ethnicity (e.g., if a person had 5R/5R on DAT1, then he/she was 83 % likely to be Black). No individuals reported more than two ethnicities. Three individuals were excluded for reporting prior strokes or seizures. In addition, four subjects were excluded for experimenter error. The final data set contained data from 146 individuals who had complete behavioral data.

Subjects reporting ADHD were not automatically excluded because attention symptoms exist in a continuum across the general population [49–51]. However, models including and excluding these subjects were compared to verify that these subjects were not driving the results. We determined that the same statistical decisions would have been reached except for slight differences in *p* values obtained, so we retained the subjects reporting ADHD. Eight subjects in the final data set reported a diagnosis of ADHD, four of whom were medicated (one additional subject reporting ADHD was excluded for having fewer than 200 trials). However, fewer than half of these eight subjects had the high risk genotype on any given genetic marker so we reasoned that these subjects could not drive any results consistent with an analysis by risk allele. Ninety-four of 107 subjects in the Rice University sample had data that met inclusion criteria (45.74 % male) as did 51 of the 54 community subjects (41.18 % male). Overall, 90.06 % of the subjects had data that was not excluded. The mean age for the university sample was 20.22 years (range 18–38 years), and for the community sample the mean age was 35.45 years (range 18–61 years).

For behavioral data collection, subjects completed a choice response task (responding with a button press to indicate either a left or right target). Subjects viewed a 1024 × 768 pixel CRT monitor with a background luminance of 0.08 cd/m^2^. A fixation cross, centered on the monitor, was always visible. Subjects were instructed to fixate the central cross and to maintain fixation throughout data collection. Before beginning data collection trials, subjects were dark adapted and completed 20 practice trials.

**Fig. 2:**
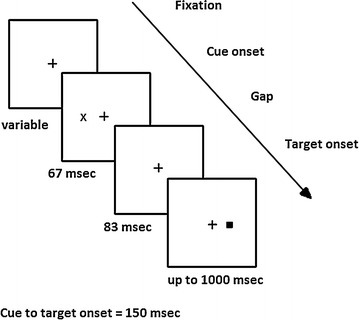
Representation of stimuli. The pre-cue stimulus flashed on for 67 ms and then off for 83 ms. Targets were on the same side as the single cue 50 % of the time. The target remained on display until a response was made but for no longer than 1000 ms. Dual cues were identical except that two cues appeared simultaneously in the same locations in which the single cues appeared

One or two pre-cues were flashed for 67 ms. On all except “catch” trials (for which no target appeared), there was an 83 ms gap after the offset of the cue(s) and prior to the onset of the target. The target remained on display for 1000 ms or until the subject made a key press (see Fig. 2). Subjects were asked to respond as quickly as possible while maintaining accuracy by making a key press to indicate a target either to the left (pressing ‘A’ on the left side of the keyboard) or to the right (pressing ‘L’ on the right side of the keyboard) of fixation. On catch trials, no target appeared, and subjects were instructed to withhold responding. After the subject responded (or the trial timed-out), there was a variable delay (1.3–1.8 s), and the next trial began. The task did not provide feedback.

In a traditional Posner-like reflexive orienting task, there are only single pre-cues (except for a neutral condition used as a baseline, which uses two equiluminant cues). To these we added unequal-luminance (“asymmetric”), dual-cue trials [52] in order to examine the ability to benefit from either of two simultaneous pre-cues with possibly different saliences. As in a traditional Posner-like task, the cues could be valid (i.e., the target would subsequently appear ipsilateral to the cue location) or invalid (i.e., contralateral). For dual cues, the target could appear either near the brighter of the two cues or near the dimmer. Never more than one target was presented and we averaged the RTs for left and right target presentations. Having, therefore, two luminances, we used a total of nine different pre-cue conditions on the display prior to the appearance of the target. These nine conditions varied on cue number (0, 1, or 2) and luminance (bright or dim). In addition, dual cues could be equiluminant or asymmetric. In this task the pre-cues were uninformative because the probability of the target appearing near where the pre-cue (or brighter cue) had appeared was 50 %.

To refer to these various cue-target conditions, we provided unique names. *Single Dim Valid* indicated a single cue of the dimmer luminance preceding an ipsilateral target. Conversely, *Single Dim Invalid* indicated a dim cue followed by a contralateral target. There were corresponding *valid* and *invalid* configurations for the *Single Bright* cues. We also included *Neutral Bright* and *Neutral Dim* cues. On these trials, identical bright or dim cues were presented simultaneously on both sides of the fixation cross. As mentioned previously, these spatially neutral cues provided baseline measures and were used to calculate alerting effects (that is, reduced RT due to the temporal appearance of the cue). When the dual asymmetric cues are presented, the target can appear either near the brighter (*Dual Asymmetric Bright*) or dimmer cue (*Dual Asymmetric Dim*). Targets can also appear uncued (the *No Cue* condition). RT was measured from the onset of the target. Finally, there was a tenth condition for which we do not have RT data because no target appeared (*Catch* trials) and subjects were instructed to withhold responding. Each of the 10 conditions (nine target-present plus one target-absent) was presented 20 times (10 times with a left target and 10 times with a right target), yielding 200 trials.

Subjects were told (1) that the cues did not predict the target’s location and (2) to ignore the cues as much as possible. Subjects completed all trials within one session with pauses as necessary.

The brighter and dimmer cue luminances were 11.7 and 2.0 cd/m^2^, respectively. The target (a square) always had a luminance of 15.5 cd/m^2^. The centermost edge of the target appeared 5.5° to either side of the fixation cross. The cues were shaped like the letter X, measured 0.8 (width) × 1.0 (height) degrees, and appeared 7.3° (innermost edges) to the left and right of the display’s center.

### Genetic methods

Saliva was collected using Oragene-250 kits (DNA Oragene, Kanata, Ontario, Canada). Genetic assays were performed to identify genotypes at known SNPs (see Table 15) using polymerase chain reaction (PCR) amplification. Purification was carried out on products by loading them onto an Applied Biosystems 3730xl DNA analyzer and reading the results using Mutation Surveyor software (SoftGenetics, PA, USA). For more details, please see our earlier paper [1].

**Table 15:** The nucleotide sequences (“primers”) used to isolate the polymorphisms analyzed

Polymorphism	Strand	Primer sequence
rs429358 (*APOE*)	Sense	5′-GAACTGGAGGAACAACTGAC
	Antisense	5′-CGCTCGCGGATGGCGCTGA
rs7412 (*APOE*)	Sense	5′-GAACTGGAGGAACAACTGAC
	Antisense	5′-CGCTCGCGGATGGCGCTGA
rs4680 (*COMT*)	Sense	5′-GCTACTCAGCTGTGCGCATG
	Antisense	5′-ACGTGGTGTGAACACCTGGT
SL6A3 repeat (*DAT1*)	Sense	5′-TGTGTGCGTGCATGTGG
	Antisense	5′-GCTTGGGGAAGGAAGGG
rs1108580 (*DBH*)	Sense	5′-ACGCCTGGAGTGACCAGAAG
	Antisense	5′-CCATCCTCCTTGGCTTTCTC
rs747302 (*DRD4*)	Sense	5′-CGGAGGGAATGGAGGAGGGA
	Antisense	5′-AGACCTGAGCTCAGGCTCTG

Genetic analysis of the VNTR (*DAT1* exon 8) was performed using fluorescently labeled PCR products (see Table 13). Similar to the SNPs, the amplified VNTR products were analyzed on an Applied Biosystems 3730xl DNA analyzer. Genemapper 4.0 software was used to assign the allele distribution (Applied Biosystems).

The potential for spurious associations in genetics studies, termed population stratification, is a commonly discussed problem [53]. Stratification in this case is an artifact that occurs when there are systematic differences in a phenotype that have nothing to do with a genetic marker under study, yet the association appears statistically significant. Spurious relationships are possible because ethnicity is related to genetic ancestry. Studies are more at risk for these stratification artifacts when subjects from various ethnic groups are (1) combined in the same analysis, (2) differ on a phenotype, and (3) simultaneously differ for unrelated reasons on the frequencies of target genotypes. In a particular study it is often impossible to determine if ethnic group phenotypic differences are due to genetic differences. However, population stratification is often suspected if a study fails to replicate. To address potential stratification, we used a strategy similar to that recommended by Hutchison et al. [54]. That is, we used self-reported ethnicity as a proxy for genetic subpopulation entered as a covariate in the statistical model.

We examined markers on five genes, *COMT*, *DAT1*, *DBH*, *DRD4*, and *APOE*. The first four each have three genotypes based on two alleles (one inherited from each parent, such as AA, AG and GG for *COMT*). All genes except *APOE* were coded ordinally so that “0” represents no risk alleles, “1” represents one risk allele, and “2” represents two risk alleles. *APOE* was coded so that “0” indicates possession of a protective allele, “1” represents the most common “normal” variant, and “2” represents possession of either one or two risk alleles.

The coding for *APOE* has a slightly different interpretation because it consists of two different markers (a haplotype) in contrast to the other genes that each had only one marker. However, the risk for cognitive deficits appears to be additive and can still be tested for linear effects using similar coding. *APOE* is based on two SNPs from which we have created three groups (ε2/ε3, ε3/ε3, and any ε4) based on the literature. For example, Hubacek et al. [55] did not consider individuals with the ε2/ε4 genotype since one allele carries risk for cognitive deficits (even in middle-aged adults without Alzheimer’s) [31] and the other provides protection against cognitive deficits [56]. In our data set two individuals had the ε2/ε4 genotype and were therefore excluded from analysis on this gene. Other genotypes, such as ε2/ε2, are rare and do not occur in our data set. The first group (ε2/ε3) represents those with a protective allele; the second group represents those with typical risks, and the third group represents those with at least one risk allele (that is, either one or two ε4 alleles) since even one risk allele carries risk for cognitive deficits. These five genes (*COMT*, *DAT1*, *DBH*, *DRD4*, and *APOE*) were entered as potential predictors of RT variability for each cue-target condition in turn.

### Statistical analysis

Prior to the main analyses, we determined that assumptions of normality were not met (the RT variables had significant positive skew). Raw RT values were transformed using a base ten logarithm. We did not use incorrect (wrong side) or RT out of range trials, and this reduced the impact of attentional lapses on slope.

Recall that 16 subjects had their data removed for reasons described under Subjects and Data Set. In addition to these exclusions, some subjects had missing genetic information due to the inability to obtain genotypic information from their saliva sample on a particular marker. For *APOE*, 12 subjects had missing genetic information. For *COMT*, two subjects had missing genetic information. For *DAT1*, seven subjects had missing genetic information. For *DBH*, three subjects had missing genetic information. Finally, for *DRD4* the number of subjects with missing genetic information was 13. If subjects were missing genotyping data for a particular marker, they were excluded from that analysis.

We used a multilevel modeling approach in SPSS (IBM, 2012) using the MIXED command. We used a backwards design similar to backwards regression. There were nine final models (one for each cue-target condition). Final models were determined by comparison to an empty model with no predictors and to simpler models. Essentially, the multi-level model is fitting a line for each person through the log transformed RTs of each cue-target condition and then the interaction between trial and genotype indicates if the slope changes by genotype. Epworth sleepiness scores, age, and ethnicity were entered as covariates.

We examined slope using mixed (multilevel) modeling. Multilevel modeling accounts for the nested nature of the data (i.e., that trials can be attributed to individuals). We used this method primarily to account for the cross-level interaction that we were interested in between trial and genotype (i.e., the slope) [57]. In addition, the intraclass correlation indicated that 4.39 % of the variance came from the individual-level data (level-2). Although this is relatively small, using multilevel modeling addresses the nested nature of the data. We entered all five genetic markers, sleepiness, age, side of target (right or left), trial number, standard deviation of base10 logarithm-transformed RT, error rate, and ethnicity (dummy coded). We also entered the interaction between trial number and genetic marker as predictors of RT. These were all fixed effects. Trial was entered as a repeated effect. The output of interest when examining RT slopes over the course of a task is the interaction between the genetic marker and trial number.

## Footnotes

1 The parameter estimates shown in Tables 3, 4, 5, 6, 7, 8, 9, 10 and 11 have been retransformed. The statistical models were run on the log(10) transformed RT data. For ease of exposition, we took each of these parameter estimates and retransformed them so that their magnitudes would be interpretable in terms of the original, untransformed scale that used RT in msec. Note that it is not appropriate simply to antilog(10) these parameter estimates; that is, it is not appropriate to compute the retransformed value of the parameter β_1_ as 10^β1^. Instead, we used the formula shown below to retransform these parameters estimates [58]. Given a parameter, β_1_, estimated using the log(10) transformed values of RT as the outcome variable, the retransformed value of this parameter estimate is:

$$b_{1} = 10^{{\widehat{{\left( {\log_{10} \left( {RT} \right)} \right)}}}} \left( {\beta_{1} \frac{1}{N}\mathop \sum \limits_{i = 1}^{N} 10^{{e_{i } }} } \right)$$

where *b*_*1*_ is the transformed parameter estimate, *β*_*1*_ is the original estimate using log(10) transformed RT, $\widehat{{{ \log }_{10} (RT)}}$ is the mean of the log(10) transformed RT values, and the summation/averaging expression after β_1_ is the mean of the antilogged residuals.

2 Tables 3, 4, 5, 6, 7, 8, 9, 10 and 11 with the original parameter estimates, i.e., the estimates before they were retransformed to the original scale, are available from the first author upon request.

3 The names for the various conditions are described in the “Methods”.
